# YC-1 sensitizes the antitumor effects of boron neutron capture therapy in hypoxic tumor cells

**DOI:** 10.1093/jrr/rraa024

**Published:** 2020-05-05

**Authors:** Takaomi Harada, Katsumi Hirose, Yuki Wada, Mariko Sato, Koji Ichise, Masahiko Aoki, Takahiro Kato, Ken Takeda, Yoshihiro Takai

**Affiliations:** 1 Department of Radiation Oncology, Southern Tohoku BNCT Research Center, 7-10 Yatsuyamada, Koriyama, Fukushima 963-8052, Japan; 2 Course of Radiological Technology, School of Health Sciences, Tohoku University School of Medicine, 2-1 Seiryo-cho, Aoba-ku, Sendai, Miyagi 980-8575, Japan; 3 Department of Radiology and Radiation Oncology, Hirosaki University Graduate School of Medicine, 5 Zaifu-cho, Hirosaki, Aomori 036-8562, Japan; 4 Department of Radiology, Akita University Graduate School of Medicine, 1-1-1 Hondo, Akita, Akita 010-8543, Japan; 5 Preparing Section for New Faculty of Medical Science, Fukushima Medical University, 1 Hikarigaoka, Fukushima, Fukushima 960-1295, Japan

**Keywords:** boron neutron capture therapy, hypoxia, deferoxamine, hypoxia-inducible factor 1α, YC-1

## Abstract

The uptake of boron into tumor cells is a key factor in the biological effects of boron neutron capture therapy (BNCT). The uptake of boron agents is suppressed in hypoxic conditions, but the mechanism of hypoxia-induced modulation of suppression of boron uptake is not clear. Therefore, we evaluated whether hypoxia-inducible factor 1α (HIF-1α) contributes to attenuation of the antitumor effects of BNCT in hypoxic tumor cells. We also tested whether YC-1, a HIF-1α-targeting inhibitor, has therapeutic potential with BNCT. To elucidate the mechanism of attenuation of the effects of BNCT caused by hypoxia, deferoxamine (DFO) was used in experiments. Cells were incubated in normal oxygen, hypoxic conditions (1% O_2_) or 5 μM DFO for 24 h. Then, cells were treated with ^10^B-boronophenylalanine (BPA) for 2 h and boron accumulation in cells was evaluated. To clarify the relationship between HIF-1α and L-type amino acid transporter 1 (LAT1), gene expression was evaluated by a using HIF-1α gene knockdown technique. Finally, to improve attenuation of the effects of BNCT in hypoxic cells, BNCT was combined with YC-1. Boron uptake was continuously suppressed up to 2 h after administration of BPA by 5 μM DFO treatment. In cells treated with 5 μM DFO, LAT1 expression was restored in HIF-1α-knocked down samples in all cell lines, revealing that HIF-1α suppresses LAT1 expression in hypoxic cells. From the results of the surviving fraction after BNCT combined with YC-1, treatment with YC-1 sensitized the antitumor effects of BNCT in cells cultured in hypoxia.

## INTRODUCTION

Boron neutron capture therapy (BNCT) is a unique treatment technique in which cancer cells are damaged by alpha particles (^4^He) and ^7^Li nuclei, which are both high linear energy transfer particles derived from the nuclear transmutation reaction of ^10^B and thermal neutrons. The path lengths of these high linear energy transfer particles are about 9 and 5 μm, respectively, which are almost the same as the tumor cell diameter. Therefore, if ^10^B accumulates selectively in cancer cells, theoretically, only cancer cells could be destroyed without damaging surrounding normal cells. *p*-Boronophenylalanine (BPA) is generally used as a boron agent for BNCT. For effective antitumor activity, 20 ppm or more ^10^B must be taken up into tumor cells [1]. Thus, a large amount of boron drug must be administered to the patient. This can be achieved because BPA has almost no pharmacological effect or toxicity. Damage to normal tissue adjacent to the tumor in the irradiation field is relatively low compared to photon therapy and particle beam therapy, depending on tumor cell selectivity for BPA [2–4]. Therefore, BNCT is considered less burdensome on heavily pretreated cancer patients. Especially for recurrent tumors following radiotherapy, re-irradiation with conventional radiotherapy brings a significant risk, as the cumulative dose of surrounding normal tissue has already reached near the tolerable dose by first radiotherapy. However, BNCT is considered to be relatively safe for re-irradiation because it can minimize the dose to normal cells adjacent to tumors, depending on low boron uptake [5]. Therefore, recurrent glioblastoma and recurrent head and neck cancer after standard treatment are considered good indications for BNCT.

^10^B-BPA, which is used most frequently in BNCT, is a chemically modified phenylalanine with a boronic acid residue that is taken up by cancer cells through the L-type amino acid transporter 1 (LAT1), which mediates retrograde transport of other amino acids [6]. When treating patients, administration is started 2 h before neutron irradiation, and BPA selectively accumulates in tumor cells via LAT1. The boron concentration in blood is maintained at 20-40 ppm and irradiation is then performed. ^18^F-BPA, in which ^18^F is added to BPA, accumulates in tumors identically to BPA. Recently, a 4-borono-2-^18^F-fluoro-phenylalanine positron emission tomography (PET) scan was performed to evaluate approximately whether ^10^B-BPA accumulates in the tumor before BNCT [7, 8].

Tumors have hypoxic regions with poor oxygen supply due to abnormal angiogenesis and rapid growth leading to insufficient blood flow [9]. In hypoxic tumor cells, hypoxia-inducible factor 1α (HIF-1α) accumulates as an adaptive response to hypoxia. As a result of gene expression that is regulated by HIF-1α, changes that promote tumor growth, such as adaptation of nutrient metabolism, hyperangiogenesis and enhancement of infiltration ability, occur [10, 11], and resistance to therapeutics such as chemotherapeutic agents and molecular targeted agents is acquired.

A few papers have reported the effects of hypoxia on BNCT treatment. BPA uptake into tumor cells incubated in chronic hypoxic conditions is suppressed significantly depending on the oxygen concentration [12, 13]. Thus, hypoxia may suppress LAT1 expression, resulting in suppression of BPA uptake into tumor cells and attenuation of the antitumor effects of BNCT. If the molecular mechanism of hypoxia-induced modulation of LAT1 expression is clarified, new therapeutic targets may be developed that enhance the treatment effect of BNCT. Therefore, in this study, we investigated whether HIF-1α contributes to attenuation of the antitumor activity of BNCT and evaluated whether inhibitors targeting HIF-1 have therapeutic potential.

## MATERIALS AND METHODS

### Materials

Deferoxamine (DFO) and 3-(5′-hydroxymethyl-2′-furyl)-1-benzylindazole (YC-1) were obtained from Abcam (Cambridge, UK). Dimethyl sulfoxide was obtained from Sigma-Aldrich (St. Louis, MO, USA).

### Cell culture and hypoxic conditions

**Fig. 1.**
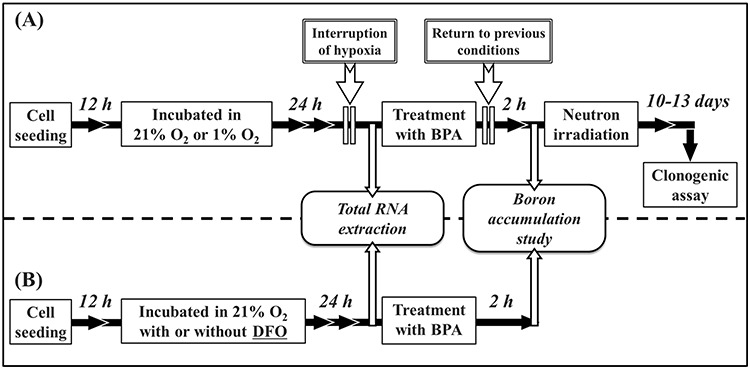
Study protocol. Cells were seeded and incubated for 12 h in (**A**) 21% O_2_ (normal oxygen conditions) or 1% O_2_ (hypoxic conditions), and (**B**) normal oxygen conditions with or without DFO for 24 h. In both time courses, after incubation for 24 h, total RNA was extracted and first-strand cDNA was synthesized. LAT1 expression was assessed by qRT-PCR. In the time course in (A), BPA was added to the culture medium with short-term interruption of the hypoxic condition, and then returned to the previous condition. After treatment with BPA for 2 h, boron accumulation was measured by inductively coupled plasma atomic emission spectroscopy. To evaluate the surviving fraction, after treatment with BPA for 2 h, cells were exposed to neutron beams. After neutron irradiation, cells were resuspended by trypsinization, plated onto dishes, cultured for 10-13 days and then used for the clonogenic assay.

The human glioblastoma cell line T98G, human oral squamous cell carcinoma cell line HSC-3 and human breast adenocarcinoma cell line MCF-7 were provided by the Cell Resource Center for Biomedical Research, Institute of Development, Aging and Cancer, Tohoku University (Sendai, Japan). T98G cells were cultured in serum-free Dulbecco’s modified Eagle’s medium/Ham’s nutrient mixture F-12 (DMEM/F12 1:1; FUJIFILM Wako Pure Chemical, Osaka, Japan), and HSC-3 and MCF-7 cells were cultured in serum-free DMEM (FUJIFILM Wako Pure Chemical). All media were supplemented with 10% fetal bovine serum (Sigma-Aldrich) and 1% penicillin/streptomycin (Gibco Life Technologies, Waltham, MA, USA), and cells were maintained at 37°C in a 5% CO_2_ atmosphere. Hypoxic conditions were achieved by culturing cells in modular incubator chambers (Billups-Rothenberg Inc., Del Mar, CA, USA). The chambers were flushed with mixed gas (95% N_2_ and 5% CO_2_) to achieve hypoxic conditions (1% O_2_). After flushing, the oxygen concentration was confirmed using a JKO-02 Ver. III oxygen monitor (JIKCO, Tokyo, Japan), and the chambers were sealed and incubated at 37°C. Cells were seeded at a density of 3.0–5.0 × 10^5^ cells depending on the growth rate, and after 12 h, cells were incubated in normal oxygen (21% O_2_) or hypoxic conditions. The study protocol is shown in Fig. 1.

**Fig. 2.**
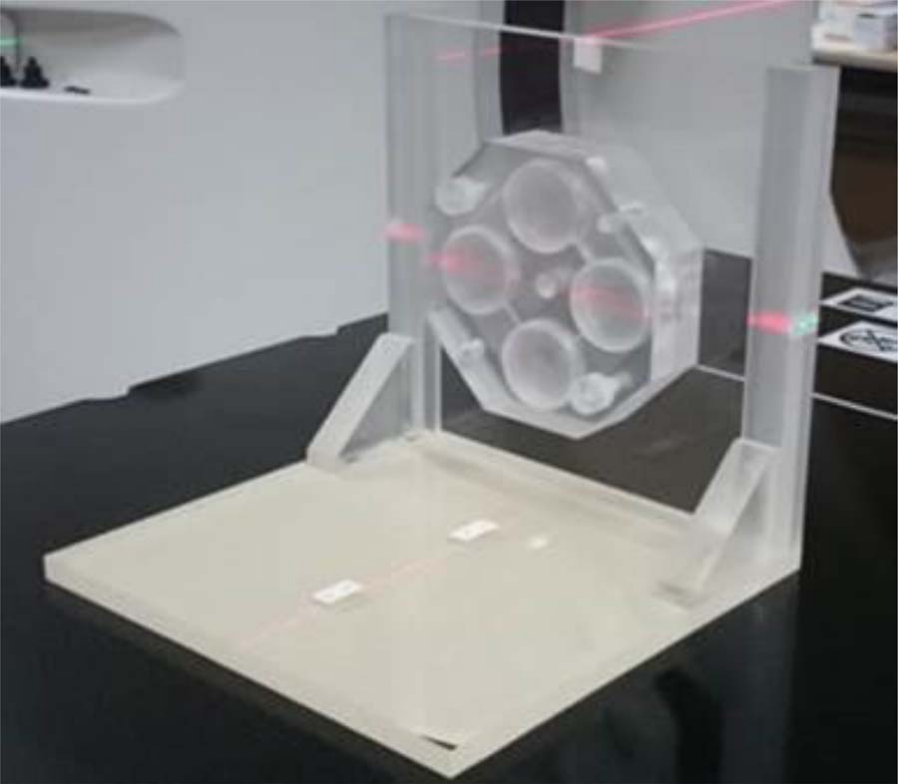
The acrylic phantom for cell irradiation. This phantom was made of 2-cm-thick acrylic plates and can be set on four cell culture dishes of 35 mm in diameter at a time. In neutron irradiation, this phantom was set as close as possible on the irradiation port.

### Trypan blue dye exclusion test

Cells were plated into 24-well plates at 1.0 × 10^5^ cells/well and cultured with each DFO and YC-1 concentration. After 24 and 48 h of incubation, cells were stained using 0.5% Trypan Blue Stain Solution (Nacalai Tesque, Inc., Kyoto, Japan) and cell viability was assessed by counting the number of unstained cells with a hemocytometer.

**Fig. 3.**
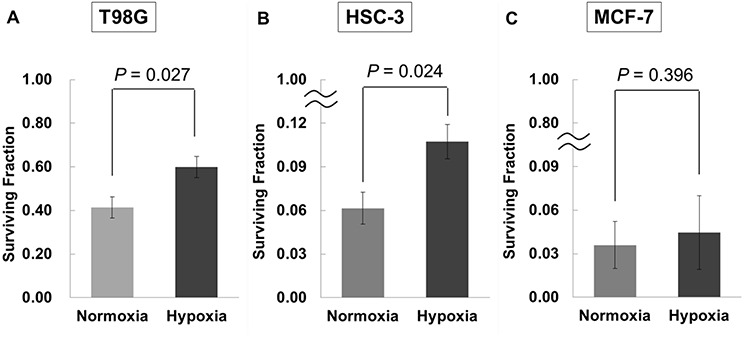
Surviving fraction of hypoxic cells after neutron irradiation. (**A**) T98G, (**B**) HSC-3 and (**C**) MCF-7 cells were incubated in normal oxygen conditions or hypoxia for 24 h, and then treated with BPA for 2 h. Then, cells were exposed to neutron beams, resuspended by trypsinization and plated onto dishes. After culturing for ~10 days, the clonogenic assay was performed. The results were normalized to normal oxygen conditions and are presented as the mean ± standard error of three different experiments. The results of the surviving fraction of each cell line show that the antitumor effects of BNCT were attenuated in hypoxic cells.

### Fluorescence imaging

To evaluate the intracellular oxygen state in a viable cell, fluorescence imaging was performed using a hypoxia live imaging agent, mono azo rhodamine (MAR; GORYO Chemical, Inc., Hokkaido, Japan). Cells were plated onto 8-well chamber slide (IWAKI, Tokyo, Japan) at 1.0 × 10^5^ cells/well and incubated for 24 h, then 1.0 μM MAR was added into the dishes and cells were incubated in normal oxygen conditions, hypoxic conditions or treated with 5 μM DFO in normal oxygen conditions for 6 h. After 6 h incubation under each condition, cells were stained with DNA-specific fluorescent Hoechst 33342 solution (1:100; Dojindo Laboratories, Kumamoto, Japan) and incubated for 15 min at 37°C in the dark. The observation of fluorescent signals was performed using an SP8 LIGHTNING Confocal Microscope (Leica Microsystems).

### Transfection with small interfering RNA

Cells were plated onto 35-mm dishes at 3.0 × 10^5^ cells/dish and incubated for 12 h. Then, 1 μL of small interfering RNA (siRNA) targeting human HIF1A (MISSION® siRNA; Sigma-Aldrich; Merck KGaA) was diluted in 50 μL Opti-MEM® I Reduced Serum Medium (Gibco; Thermo Fisher Scientific, Inc.) and mixed with 3.5 μL of Lipofectamine® RNAiMAX Reagent (Invitrogen; Thermo Fisher Scientific, Inc.) pre-diluted in 50 μL of Opti-MEM® I Reduced Serum Medium. After 20 min of incubation at room temperature, the complexes were added to the cells in a final volume of 1 mL of medium and cells were incubated for 24 h. As a control for siRNA, BLOCK-iT™ Alexa Fluor® Red Fluorescent Oligo 20 μM (Invitrogen; Thermo Fisher Scientific, Inc.) was used. After incubation for 24 h, cells were washed with Dulbecco’s phosphate-buffered saline (FUJIFILM Wako Pure Chemical), dissociated by trypsinization and plated onto new 35-mm dishes at 3.0 × 10^5^ cells/dish. After 24 h, 5 μM DFO was added to the culture medium and cells were incubated for 24 h.

### Total RNA extraction and quantitative real-time polymerase chain reaction

Total RNA was extracted using NucleoSpin® RNA (Takara Bio Inc., Otsu, Shiga, Japan) according to the manufacturer’s protocol. First-strand cDNA was synthesized with an iScript RT Supermix for RT-qPCR® (Bio-Rad, Hercules, CA) from extracted total RNA according to the manufacturer’s protocol. Gene expression was assessed using quantitative real-time polymerase chain reaction (qRT-PCR) with SsoAdvanced Universal SYBR Green Supermix® (Bio-Rad), with typical amplification parameters (95°C for 30 s followed by 60 cycles at 98°C for 10 s and 60°C for 30 s) using a CFX connect™ real-time PCR detection system (Bio-Rad). Relative differences were determined with the crossing-point method with a standard curve. mRNA expression was determined after normalization to the housekeeping gene glyceraldehyde-3-phosphate dehydrogenase (GAPDH). To evaluate the possibility of changes in gene expression of GAPDH under hypoxia, the cDNA concentration of each sample after the reverse transcription reaction of extracted total RNA was normalized using Qubit 4 Fluorometer (Invitrogen; Thermo Fisher Scientific, Inc.) and then RT-PCR was performed. The oligonucleotide primer sets used for qRT-PCR purchased from Takara Bio Inc. are shown in Table 1.

**Table 1:** Primers used for quantitative real-time PCR

Gene sequence	Primer
GAPDH	F: 5′-GCACCGTCAAGGCTGAGAAC-3′
	R: 5′-TGGTGAAGACGCCAGTGGA-3′
SLC7A5 (LAT1)	F: 5′-GCATCGGCTTCACCATCATC-3′
	R: 5′-ACCACCTGCATGAGCTTCTGAC-3′
HIF1A	F: 5′-CTCATCAGTTGCCACTTCCACATA-3′
	R: 5′-AGCAATTCATCTGTGCTTTCATGTC-3′

F: Forward, R: Reverse.

### Western blot analysis

Cells were incubated in 35-mm dishes at a density of 5.0 × 10^5^ cells. After 12 h, cells were incubated in hypoxic conditions or treated with 5 μM DFO in normal oxygen conditions for 24 h. Cells were lysed using Cell Lysis Buffer (cat. no. 9803 s; Cell Signaling Technology, Inc., Danvers, MA, USA) and protease inhibitor cocktail (cat. no. 635673; Takara Bio Inc.). Cell lysates and pre-stained molecular weight markers were separated by sodium dodecylsulfate polyacrylamide gel electrophoresis with 12% Mini-PROTEAN® TGX™ precast gels (Bio-Rad Laboratories, Inc.) and then transferred onto polyvinylidene fluoride membranes with Trans-Blot® Turbo™ (Bio-Rad Laboratories, Inc.). The membranes were blocked with Block ACE® (cat. no. UK-B80; DS Pharma Biomedical Co., Ltd., Osaka, Japan) dissolved in distilled water and then incubated with various primary antibodies diluted in blocking buffer Can Get Signal® solution 1 (Can Get Signal® Immunoreaction Enhancer Solution; Toyobo Biochemicals, Osaka, Japan). Membranes were incubated with mouse monoclonal anti-actin antibody (cat. no. sc-47778; 1:1,000; Santa Cruz Biotechnology, Dallas, TX, USA) and mouse monoclonal anti-HIF-1α antibody (cat. no. 610959; 1:500; BD Biosciences) for 1 h. The blots were then washed three times with Tris-buffered saline containing 0.1% Tween-20 (Bio-Rad Laboratories, Inc.) and incubated with anti-mouse IgG, horseradish peroxidase-linked antibody (cat. no. 7076; 1:2,000; Cell Signaling Technology, Inc.) in blocking buffer Can Get Signal® solution 2 (Can Get Signal® Immunoreaction Enhancer Solution; Toyobo Biochemicals) for 1 h. Membranes were washed three times and immunoreactivity was visualized with Clarity Western ECL substrate using the chemiluminescence Molecular Imager® ChemiDoc™ XRS+ system (both from Bio-Rad Laboratories, Inc.) according to the manufacturer’s instructions.

### Boron accumulation study

^10^B-Enriched L-BPA solution and yttrium inductively coupled plasma standard solution were kindly supplied by Stella Pharma Corporation (Osaka, Japan). Cells were plated into 6-well plates at 3.0 × 10^5^ cells/well and cultured with or without 5 μM DFO for 24 h in normal oxygen conditions at 37°C. After 24 h of incubation, each sample was exposed to BPA at 30 μg ^10^B/mL in the medium for up to 2 h. After BPA treatment, the culture medium containing BPA was removed and each sample was digested with perchloric acid (HClO_4_) and hydrogen peroxide (H_2_O_2_) for 2 h at 50°C. To 500 μL of digested cell solution, 500 μL of standard yttrium solution was added and diluted with 4 mL of distilled water. ^10^B accumulation in each sample was analysed with inductively coupled plasma atomic emission spectroscopy using an ICPE-9000 (Shimadzu, Kyoto, Japan) at a wavelength of 208.956 nm. The calibration curve obtained from dilutions of the standard boron solution was linear in the range 0.05–5.0 μg ^10^B/mL.

### Neutron irradiation and clonogenic assay

Cells were cultured in normal oxygen or hypoxic conditions for 24 h and then treated with BPA for 2 h. Then, they were placed on an acrylic phantom, which was made of 2-cm-thick acrylic plates for cell irradiation and set as close as possible on the irradiation port, and exposed to neutron beams with 0.6 C as the proton charge using the accelerator-based BNCT system at Southern Tohoku BNCT Research Center. The acrylic phantom is shown in Fig. 2. The dose components of the physical dose of neutron irradiation for BNCT are shown in Table 2. After neutron irradiation, cells were resuspended with trypsinization and plated onto 60-mm dishes at 100–5000 cells/dish depending on the results of preliminary experiments. After culturing for ~10 days, the cells were fixed in 100% methanol and stained with Giemsa stain solution (Nacalai Tesque). Colonies composed of >50 cells were counted. The surviving fraction was determined by dividing the number of colonies from the irradiated culture with that of the non-irradiated control culture treated with BPA.

**Table 2:** Components of the physical dose of neutron irradiation

Neutron flux (cm^−2^)	Value
Thermal neutrons	2.36 × 10^11^
Epi-thermal neutrons	2.91 × 10^11^
Fast neutrons	1.91 × 10^10^
Dose (cGy)	Value
Boron dose (ppm^−1^)	23.18
Nitrogen dose	8.38
Hydrogen dose	27.55
Gamma dose	12.69

### Statistical analysis

All experiments except the cytotoxicity of DFO and YC-1 were performed at least three times and results are expressed as means ± standard error. Experiments for the cytotoxicity of DFO and YC-1 were performed twice and results are expressed as means ± standard deviation. Statistical significance was estimated using the Mann-Whitney U-test, Student’s t-test or Welch’s t-test. The Statcel 3 add-in (OMS Publishing, Saitama, Japan) for Microsoft Excel was used for the statistical analysis. Probability values of *P* < 0.05 were considered statistically significant.

## RESULTS

### Attenuation of the antitumor effects of BNCT under hypoxic conditions

In our previous study, hypoxic conditions with 1% O_2_ have been reported to induce a decrease of LAT1 mRNA expression in glioblastoma cell lines [12]. Therefore, at first, we evaluated the impact of hypoxia with 1% O_2_ on the toxicity of BNCT with 30 ppm ^10^B-BPA. For cells cultured in normal and hypoxic conditions and treated with 30 ppm ^10^B-BPA, the surviving fractions after neutron irradiation were 0.414 ± 0.049 and 0.600 ± 0.048 (*P* = 0.027) for T98G cells, 0.062 ± 0.011 and 0.107 ± 0.012 (*P* = 0.024) for HSC-3 cells and 0.036 ± 0.016 and 0.045 ± 0.025 (*P* = 0.396) for MCF-7 cells, respectively (Fig. 3). In agreement with previous reports, we confirmed that the antitumor effects of BNCT were attenuated in hypoxic glioblastoma and head and neck cancer cells with a potential indication for BNCT.

**Fig. 4.**
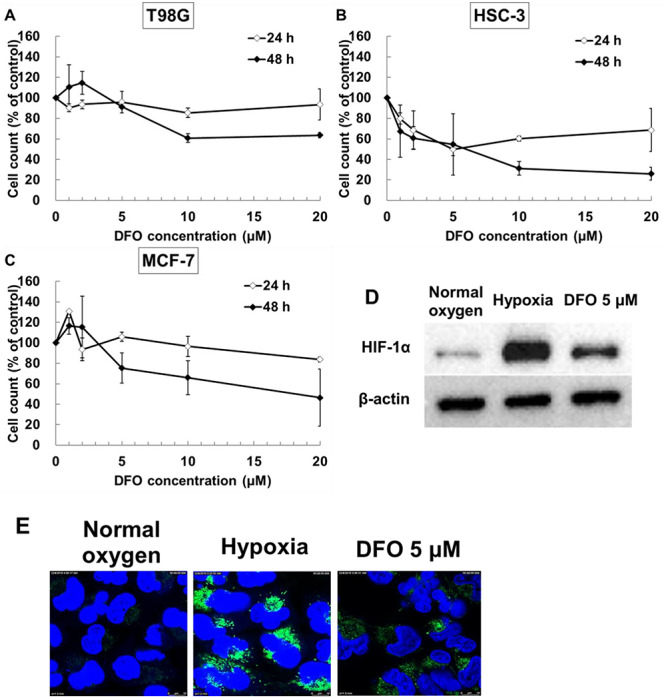
Effect of DFO as a hypoxic-mimetic agent. Cytotoxicity of DFO in (**A**) T98G, (**B**) HSC-3 and (**C**) MCF-7 cell lines. Cells were treated with 0-20 μM DFO. After 24 or 48 h of incubation, the trypan blue assay was performed. The results are expressed relative to non-treated samples (100%) and are presented as the mean ± standard deviation. To evaluate the pseudo-hypoxic effect of DFO on cultured cells, T98G cells were incubated in normal oxygen, hypoxia or 5 μM DFO for 24 h, and then HIF-1α protein expression (**D**) was evaluated. HIF-1α protein expression was evaluated by western blot analysis using anti-HIF-1α and anti-β-actin antibodies. β-Actin expression was used as a loading control. (**E**) Evaluation of the intracellular oxygen state in T98G cells using MAR. Cells were incubated for 24 h, then 1.0 μM MAR was added to the cells and cells were incubated under normal oxygen condition, hypoxia, or with 5 μM DFO under normal oxygen conditions for 6 h. After 6 h incubation, cells were stained with Hoechst 33342 solution to stain nuclei (blue). MAR was stained with green. Scale bar: 10 μm.

### DFO as well as hypoxia attenuates the antitumor effects of BNCT

To elucidate the mechanism of attenuation of the effects of BNCT caused by hypoxia, DFO, which is a hypoxic-mimetic agent, was used in experiments. For cytotoxicity of DFO for 24 h, the viabilities of T98G and MCF-7 cells were maintained in up to 5 μM DFO (Fig. 4A and C). In HSC-3 cells, the viability decreased according to the density of DFO (Fig. 4B). However, under treatment with 5 μM DFO, a significant increase in dead cells in HSC-3 cell lines was not confirmed for up to 48 h of incubation, and the absolute number of cells increased over time. Thus, it was considered that not much cell death, but cell growth suppression, was caused as the concentration of DFO increased. The simulated hypoxic environment induced by DFO was confirmed by enhanced expression of HIF-1α. In cells treated with 5 μM DFO, the expression of HIF-1α was enhanced compared with cells in the normal oxygen conditions (Fig. 4D). In the fluorescence imaging of hypoxic conditions in a viable cell using MAR, hypoxia was observed within a couple of hours in samples under hypoxia of 1% O_2_ and treatment with 5 μM DFO. The fluorescence intensity of DFO-treated samples was higher than that of samples under normal oxygen and lower than that of hypoxia (Fig. 4E). Thus, we confirmed that 5 μM DFO induces a pseudo-hypoxic environment.

**Fig. 5.**
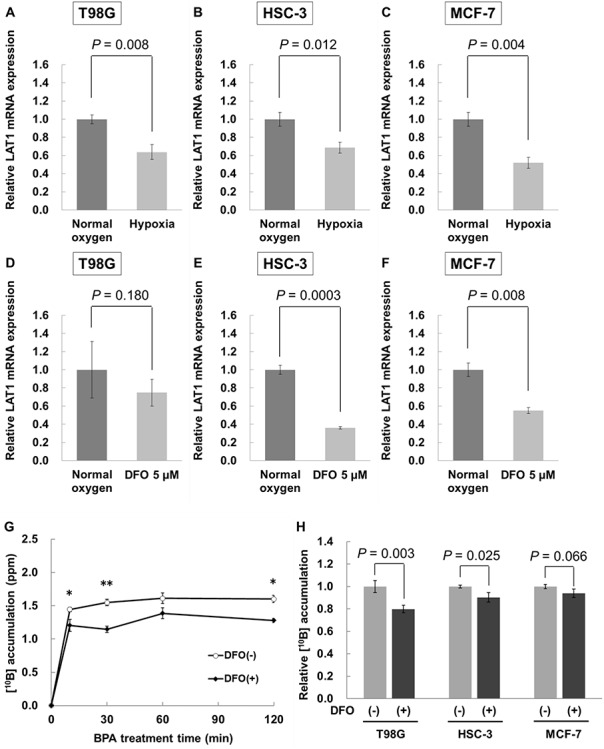
Evaluation of LAT1 expression and ^10^B accumulation under hypoxia of 1% O_2_ and treatment with 5 μM DFO. Gene expression of LAT1 in (**A**) T98G, (**B**) HSC-3 and (**C**) MCF-7 cell lines under the normal oxygen and hypoxic conditions. Gene expression of LAT1 in (**D**) T98G, (**E**) HSC-3 and (**F**) MCF-7 cell lines under the normal oxygen and 5 μM DFO treatment conditions. LAT1 gene expression was assessed using qRT-PCR. The amount of LAT1 mRNA was normalized to GAPDH mRNA. The results are shown as relative value normalized to the normal oxygen condition. (**G**) ^10^B accumulation in T98G cells. Cells were treated with 30 ppm BPA for 10, 30, 60 and 120 min after incubation in normal oxygen conditions or 5 μM DFO for 24 h. After treatment with BPA for 10, 30 and 120 min, a significant difference was observed between DFO non-treated cells and DFO-treated cells. ^*^P < 0.05 and ^**^P < 0.01. (**H**) Relative ^10^B accumulation in T98G, HSC-3 and MCF-7 cells treated with BPA for 120 min. The results are normalized to the amounts of ^10^B accumulation in DFO non-treated cells. All values except the results of cytotoxicity of DFO are presented as the mean ± standard error of three different experiments.

For gene expression of LAT1, relative LAT1 mRNA expression under hypoxia was 0.637 ± 0.082 (*P* = 0.008) for T98G cells, 0.687 ± 0.061 (*P* = 0.012) for HSC-3 cells and 0.519 ± 0.083 (*P* = 0.004) for MCF-7 cells, respectively (Fig. 5A-C). It is widely known that GAPDH is activated under hypoxic conditions in some cell lines. To evaluate GAPDH expression in hypoxic conditions, the cDNA concentration of each sample after the reverse transcription reaction of extracted total RNA was normalized, and then RT-PCR was performed. GAPDH expression made little difference in normal oxygen and hypoxia (data not shown). Therefore, according to the previous report and our study, using GAPDH as a house-keeping gene under hypoxic conditions did not seem to affect the present results. DFO-treated cells showed reduced gene expression of LAT1 [0.748 ± 0.149 (*P* = 0.180) for T98G cells, 0.360 ± 0.014 (*P* = 0.0003) for HSC-3 cells and 0.551 ± 0.034 (*P* = 0.008) for MCF-7 cells, respectively, normalized to DFO non-treated cells (Fig. 5D-F)]. Boron uptake was continuously suppressed up to 2 h after BPA addition by treatment with 5 μM DFO (Fig. 5G). The amount of intracellular boron 2 h after administration of BPA was 0.799 ± 0.033 normalized to the value in DFO non-treated cells (*P* = 0.003). In addition, boron uptake was also suppressed in HSC-3 and MCF-7 cells treated with 5 μM DFO [0.903 ± 0.043 (*P* = 0.025) for HSC-3 and 0.940 ± 0.037 (*P* = 0.066) for MCF-7, respectively] (Fig. 5H).

### Influence of HIF-1α accumulation on expression of LAT1

**Fig. 6.**
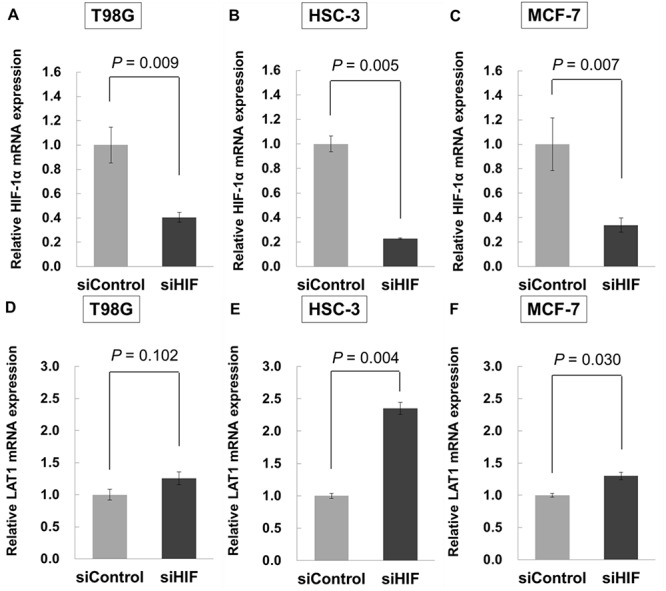
Influence of HIF-1α inhibition using siRNA. Cells were incubated for 24 h after suppressing HIF-1α expression using siRNA. To evaluate LAT1, cells were incubated for 24 h in 5 μM DFO after transfection of HIF-1α siRNA. Gene expression was assessed using qRT-PCR. HIF-1α expression is shown for (**A**) T98G, (**B**) HSC-3 and (**C**) MCF-7 cell lines, and the relative LAT1 mRNA expression is shown for (**D**) T98G, (**E**) HSC-3 and (**F**) MCF-7 cell lines. The amounts of HIF-1α and LAT1 mRNA were normalized to GAPDH mRNA. All results were normalized to samples treated with control siRNA (siControl), and are presented as the mean ± standard error of three different experiments.

HIF-1α, which accumulates intracellularly in hypoxic conditions, is a key factor in the cellular hypoxic response. To clarify the relationship between HIF-1α and LAT1, HIF-1α was knocked down with siRNA. HIF-1α was successfully reduced in all cell lines to levels that were 20–40% of that in the siRNA control conditions (Fig. 6A–C). In cells treated with 5 μM DFO, LAT1 expression was restored in all cell lines treated with siRNA (*P* = 0.102 for T98G cells, *P* = 0.004 for HSC-3 cells, *P* = 0.030 for MCF-7 cells) (Fig. 6D–F), revealing that HIF-1α suppresses LAT1 expression in hypoxic tumor cells. LAT1 expression in T98G cells transfected with HIF-1α siRNA was not so improved compared with the samples treated with control siRNA. It was suggested that the pseudo-hypoxic effect of DFO in T98G cells may have been small and accumulation of HIF-1α in cells may have been lower than in HSC-3 cells. These results suggest that HIF-1α may be a therapeutic target for enhancing the antitumor effects of BNCT in tumors with hypoxic fractions.

**Fig. 7.**
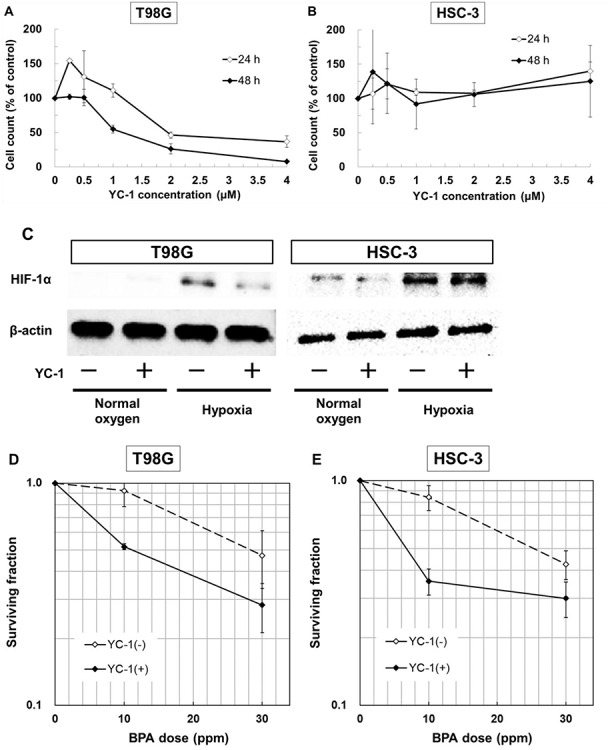
Effect of the HIF inhibitor YC-1 as a sensitizer of hypoxic cells to BNCT. Cytotoxicity of YC-1 in (**A**) T98G and (**B**) HSC-3 cells. Cells were treated with 0-4 μM YC-1, and after 24 and 48 h of incubation, the trypan blue assay was performed. The results are expressed relative to non-treated samples (100%) and are presented as the mean ± standard deviation. (**C**) HIF-1α protein expression in T98G and HSC-3 cells incubated in normal oxygen and hypoxia with or without 0.5 μM YC-1 for 24 h. HIF-1α protein expression was evaluated by western blot analysis using anti-HIF-1α and anti-β-actin antibodies. β-Actin expression was used as a loading control. The surviving fraction after treatment with YC-1 and neutron irradiation for (**D**) T98G and (**E**) HSC-3 cells. Cells were irradiated after treatment with 0.5 μM YC-1 and incubation in hypoxia for 24 h followed by treatment with 10 or 30 ppm BPA for 2 h. The results are presented as the mean ± standard error of more than three different experiments.

### The HIF inhibitor YC-1 sensitizes the antitumor effects of BNCT in hypoxic tumor cells

YC-1 at 0.5 μM or lower showed no toxicity (Fig. 7A and B). In T98G cells treated with 0.5 μM YC-1, HIF-1α protein expression after 24 h incubation under hypoxia decreased compared with YC-1 non-treated cells. In HSC-3 cells, HIF-1α protein expression of 0.5 μM YC-1-treated samples slightly decreased (Fig. 7C). The surviving fraction after neutron irradiation in cells treated with BPA in the presence or absence of YC-1 is shown in Fig. 7D and E. Treatment with YC-1 sensitized the antitumor effects of BNCT in both T98G and HSC-3 cells cultured in hypoxic conditions.

## DISCUSSION

To elucidate the mechanism of attenuation of the antitumor effects of BNCT in hypoxic conditions, we created a simulated hypoxic environment using DFO. DFO, which is clinically used as an iron chelator for treatment of patients with iron overload disease, inhibits the activity of prolyl hydroxylase, suppresses the transcriptional activity of HIF-1α and stabilizes HIF-1α [14]. The advantage of using DFO is that a hypoxic state can be created easily by simply adding DFO to the cell culture. In addition, construction of a pseudo-hypoxic condition using DFO *in vitro* has already been performed in many previous studies, and therefore, treating cultured cells with DFO for analysis of hypoxia in this study is appropriate [15, 16]. On the other hand, the disadvantage of DFO is that the intracellular oxygen state induced by DFO is not known. Furthermore, the chelating effect of DFO and the hypoxia load in cultured cells may produce different effects on organelles. However, evaluation of the HIF-1α protein expression level showed a similarity between pseudo-hypoxic conditions induced by DFO and hypoxic conditions induced by reduced oxygen (Fig. 4D). In addition, from the fluorescence imaging of hypoxic conditions using MAR, it was found that we could evaluate visually the intracellular oxygen state induced by DFO (Fig. 4E). Furthermore, regarding the gene expression of LAT1, which is involved in BPA uptake, a decrease in LAT1 expression was confirmed following DFO administration compared to normal oxygen conditions (Fig. 5D–F). Therefore, administration of DFO appears to create hypoxia-like conditions.

To clarify the relationship between HIF-1α accumulation in hypoxic cells and LAT1 expression, we evaluated the mRNA expression of HIF-1α and LAT1 after treatment with HIF-1α siRNA. In the pseudo-hypoxic condition using DFO, the gene expression of LAT1 increased in cells transfected with HIF-1α siRNA compared with the control (Fig. 6D–F). Therefore, the LAT1 expression level may recover by inhibiting HIF-1α expression. Our study showed for the first time that LAT1 expression is controlled by HIF-1α, the key factor in the cellular hypoxic response. Restoration of LAT1 expression in hypoxic cells may lead to increased boron uptake in cells and decreased cell survival after BNCT, resulting in improvement in therapeutic outcomes following BNCT. Introduction of siRNA is involved in the toxicity and the metabolism of the cell can thereby decrease, and it is suggested that BPA uptake may have been masked in both sicontrol- and siHIF-induced samples. Therefore, it was difficult to show the changes in boron concentration in HIF-1α-depleted cells.

Finally, we evaluated the possibility of sensitization of cells to the therapeutic effects of BNCT by using a HIF inhibitor in hypoxic conditions. It was confirmed that the gene expression of LAT1 recovered under HIF-1α knockdown conditions in all cells that we evaluated. However, in the results of the surviving fraction after neutron irradiation for hypoxic cells treated with BPA, a meaningful difference was not recognized between normal oxygen conditions and hypoxia in MCF-7 cells (Fig. 3). In this study, all cell lines were irradiated under the same neutron beam conditions. Therefore, it was suggested that the sensitivity of MCF-7 cells to BNCT may have been higher than that of the other cell lines depending on cell-specific relative biological effectiveness or BPA uptake. This result might have revealed that the impact of hypoxia on BPA uptake depends on the original sensitivity to BNCT. YC-1 inhibits platelet aggregation and is used pharmacologically [17, 18]. The details of the mechanism of YC-1 are not clear but YC-1 suppresses the activity of HIF-1α in cancer cells [19], and in this study, the decrease of HIF1-α protein in YC-1-treated cells was confirmed (Fig. 7C). In addition, YC-1 has a radiosensitization effect on tumors with hypoxic fractions when used in combination with radiation therapy [20, 21]. To evaluate the hypoxic area in head and neck cancer, tumor hypoxia imaging with ^18^F-fluoromisonidazole (^18^F-FMISO) PET is useful [22–25]. Positive findings on ^18^F-FMISO PET also correlate with poor treatment outcomes following conventional radiotherapy and chemotherapy [26, 27]. Therefore, to improve the therapeutic outcomes of these tumors with hypoxic fractions, enhancing the radiosensitivity and drug sensitivity of the hypoxic region of these tumors is necessary. In this study, addition of YC-1 to glioblastoma and oral squamous cell carcinoma cell lines enhanced the cell killing effect of BNCT in hypoxic cells (Fig. 7D and E). This indicates that administration of YC-1 may enhance the therapeutic response to BNCT in patients with cancer with hypoxic fractions that have responded poorly to radiotherapy and chemotherapy.

In this study, we showed that hypoxia suppressed LAT1 expression through accumulation of HIF-1α. Concomitant use of HIF inhibitors may improve treatment resistance to BNCT for cancers containing hypoxic regions.
